# Prenatally Diagnosed Multiple Enteric Duplication Cysts in an Infant: A Rare Case and Surgical Approach

**DOI:** 10.7759/cureus.81897

**Published:** 2025-04-08

**Authors:** Elisavet Kanna, Zoi Lamprinou, Jonida Mene, Nota Giamarelou, Ioannis Skondras

**Affiliations:** 1 Pediatric Surgery, Panagiotis and Aglaia Kyriakou Children's Hospital, Athens, GRC; 2 Pediatric Surgery, Penteli Children's Hospital, Athens, GRC; 3 Histopathology, Panagiotis and Aglaia Kyriakou Children's Hospital, Athens, GRC

**Keywords:** congenital malformations, enteric duplication cyst, multiple duplication cysts, neonatal surgical management, prenatal diagnosis

## Abstract

Enteric duplication cysts (EDCs) are rare congenital anomalies that can occur along any part of the gastrointestinal tract, with multiple cysts being exceptionally uncommon. We present the case of a five-month-old infant with multiple prenatally diagnosed EDCs, including a pedunculated cyst and a large tubular duplication near the ileocecal valve. The infant remained asymptomatic, and elective surgical resection was performed. Histopathology confirmed the diagnosis, revealing distinct structural features and chronic inflammatory changes. This case highlights the morphological variety of multiple duplication cysts and underscores the importance of prenatal detection and timely surgical intervention to prevent complications.

## Introduction

Enteric duplication cysts (EDCs) are rare congenital anomalies that can develop anywhere along the gastrointestinal tract (GIT), from the mouth to the anus, with the ileum being the most common site [1]. These cysts vary in size and morphology, ranging from small, isolated lesions to larger, complex masses. Structurally, they may be tubular, running parallel to the intestine, or spherical, often located within the mesentery. Depending on their embryological origin, EDCs may or may not communicate with the adjacent bowel lumen [2].

To be classified as an enteric duplication cyst, a lesion must meet specific histological and anatomical criteria: (1) it should be contiguous with a portion of the gastrointestinal tract, (2) it must have a well-developed smooth muscle wall, and (3) it should possess an epithelial lining that resembles some part of the gastrointestinal mucosa [3]. These features distinguish EDCs from other intra-abdominal cystic lesions and are crucial for accurate diagnosis and management.

Multiple duplication cysts are particularly rare, occurring in only 1% to 7% of EDC cases. These cases can present with multiple cysts within the same GIT segment or, less commonly, across different segments, adding complexity to diagnosis and management [1,3-5]. The presence of multiple cysts increases the risk of complications, including obstruction, volvulus, perforation, hemorrhage, or infection, due to the increased likelihood of interfering with bowel function. Given the rarity, cases involving multiple EDCs present unique surgical challenges and contribute valuable insights into the pathogenesis, clinical presentations and optimal management of these anomalies.

Prenatal diagnosis of duplication cysts is uncommon, with only a few cases documented in the literature [1]. However, advances in fetal imaging, particularly high-resolution ultrasound and fetal MRI, have improved early detection. In this report, we present a rare case of multiple enteric duplication cysts diagnosed prenatally and successfully resected in infancy, highlighting the importance of early recognition and multidisciplinary management.

In this report, we present a unique case of multiple enteric duplication cysts identified prenatally and successfully resected during infancy, emphasizing the importance of early detection, surgical intervention, and postnatal follow-up in managing such rare cases.

## Case presentation

A five-month-old infant was admitted to our hospital for evaluation of two intra-abdominal cystic lesions located near the stomach. These cysts were incidentally detected during antenatal ultrasound and fetal MRI, prompting referral for further investigation and management.

On prenatal ultrasound, the lesions appeared as well-defined, anechoic, non-vascular cysts located in the upper fetal abdomen. They were unilocular, with no septations, calcifications, or solid components. Fetal MRI further characterized the cysts as T2-hyperintense, fluid-filled structures situated in close proximity to the terminal ileum and ileocecal region, without evidence of mass effect or displacement of adjacent organs. The differential diagnosis included mesenteric cysts, choledochal cysts, omphalomesenteric duct cysts, and, in female fetuses, ovarian cysts - the latter excluded based on fetal sex and anatomical location. The cysts’ close relationship to the bowel, lack of communication with the urinary or biliary systems, and their characteristic imaging features raised suspicion for enteric duplication cysts. These findings were discussed with the family, and postnatal imaging and surgical planning were recommended.

The infant was delivered at term following an uneventful pregnancy, with normal prenatal screening apart from the presence of the cystic lesions. Postnatal growth and development were within normal parameters, and the infant remained asymptomatic, with no signs of gastrointestinal obstruction, feeding intolerance, or abdominal distension.

Upon admission, a comprehensive clinical evaluation was performed. Abdominal examination revealed no palpable masses, tenderness, or signs of peritonitis. Laboratory investigations, including complete blood count (CBC), inflammatory markers, and metabolic screening, were within normal limits. A postnatal abdominal ultrasound confirmed the presence of two well-circumscribed, cystic structures, without internal vascularity, solid components, or signs of rupture. Due to the potential risks of progression, volvulus, obstruction, or secondary infection, further imaging with contrast-enhanced MRI was conducted to better characterize the lesions, determine their anatomical relationships, and support surgical planning.

The imaging findings suggested enteric duplication cysts, and a multidisciplinary team comprising pediatric surgeons, radiologists, and neonatologists discussed the optimal management approach. After detailed counseling and discussion with the infant’s parents, written informed consent was obtained for surgical intervention. Given the risk of future complications, including bowel obstruction, torsion, hemorrhage, or infection, elective surgical resection was advised to ensure definitive treatment and prevent potential morbidity.

A follow-up ultrasound was conducted prior to surgery, further delineating the cystic masses and their anatomical involvement. The decision was made to proceed with surgical resection to mitigate the risk of complications and optimize long-term outcomes.

**Figure 1:**
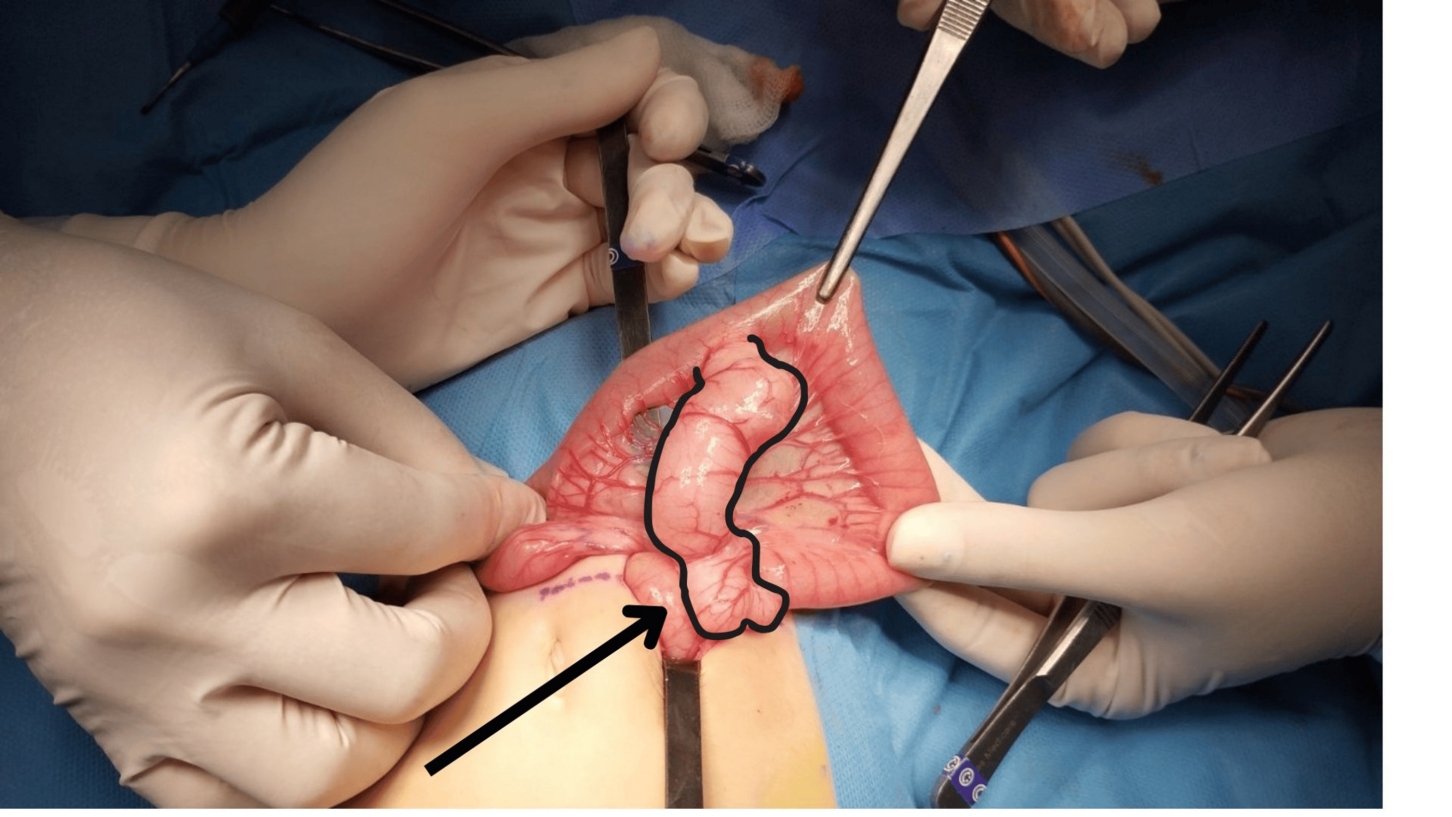
Intraoperative view showing a large tubular structure near the ileocecal valve (arrow), causing twisting of the terminal ileum without compromising its vascular integrity. The arrow highlights the duplication cyst identified during surgery.

Under general anesthesia, the peritoneal cavity was accessed, revealing an oval-shaped cystic mass at the terminal ileum, which was excised from the mesentery with no communication to the adjacent bowel segment. Additionally, a large tubular structure was observed 5 cm proximal to the ileocecal valve, causing twisting of the terminal ileum without compromising its blood supply, and exhibiting a communication with the intestinal lumen (Figure 1), suggesting an enteric duplication cyst. An enterectomy was performed, followed by a wedge-shaped end-to-end anastomosis, in which both the anterior and posterior walls were sutured to restore the continuity of the intestinal lumen. The mesenteric defect was closed to prevent internal herniation. Due to its proximity and to prevent potential secondary complications, an appendectomy was also performed.

The patient’s postoperative course was uneventful, and he was discharged on postoperative day three without complications. Follow-up evaluations showed no abnormalities, and the patient remains asymptomatic to date.

Histopathological examination of the excised specimens confirmed the presence of two distinct enteric duplication cysts: (1) A pedunculated cystic lesion measuring 1.5 × 1.3 × 1.1 cm was identified, arising from the terminal ileal mesentery. Its outer surface was smooth, with obliterated vessels, and cross-sectioning revealed a 1.1 cm cystic cavity filled with serous fluid; (2) A large tubular cystic mass, 9.7 cm in length, originated from the mesenteric rim near the ileocecal valve. It displayed a lobed configuration at one end (2 cm in diameter), transitioning into a 1.6 cm narrow segment, followed by a distended section measuring 2 cm. The terminal portion of the mass was curved and adhered to the adjacent intestinal wall, and the internal lumen, measuring 1.7 cm, contained seromucous fluid.

**Figure 2:**
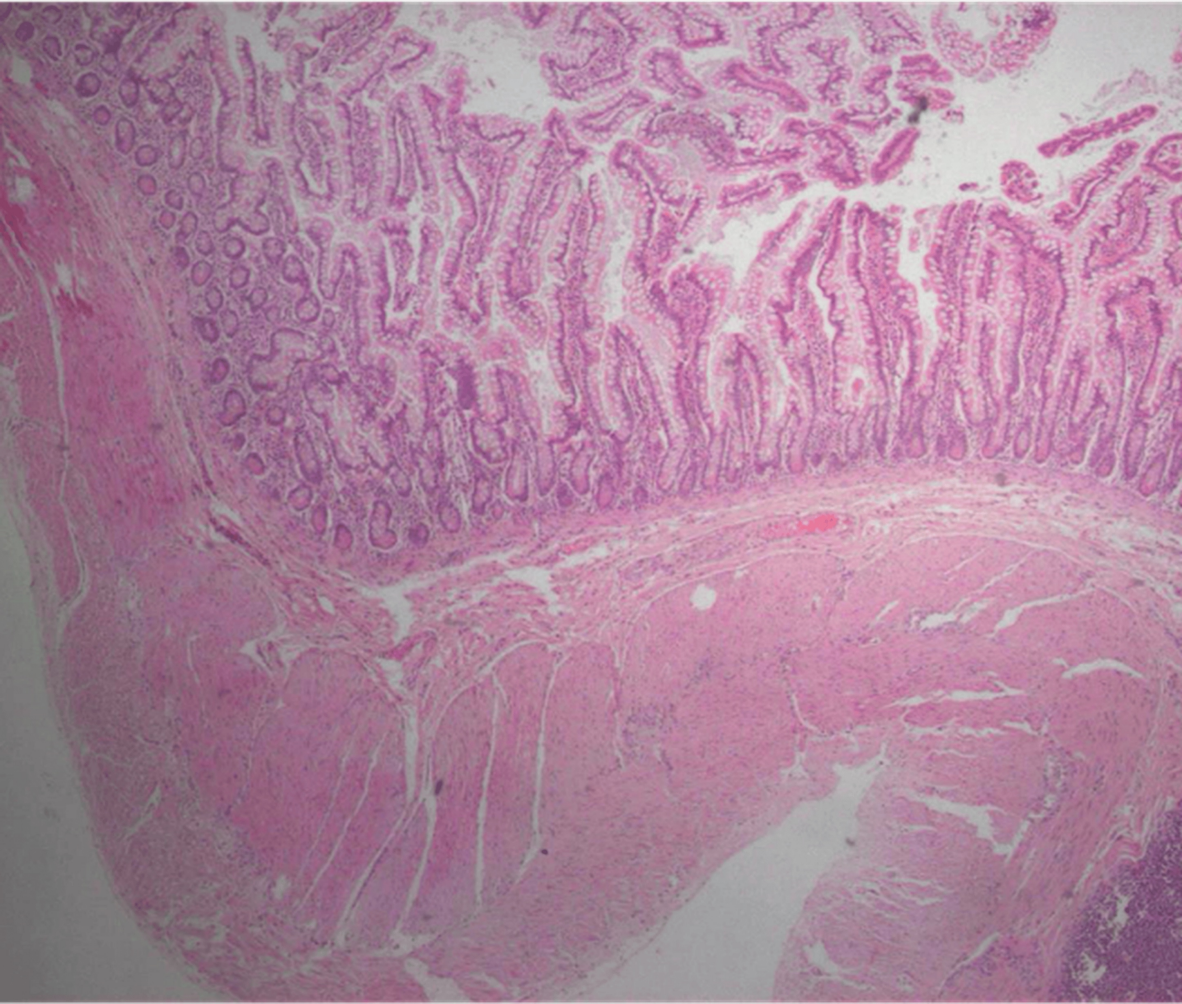
Microscopic image of the cyst wall excised from the mesentery, showing features consistent with an enteric duplication cyst. The structure resembles normal intestinal wall architecture, including mucosa, submucosa, muscularis, and serosa. Hematoxylin and eosin (H&E) stain, original magnification ×10.

**Figure 3:**
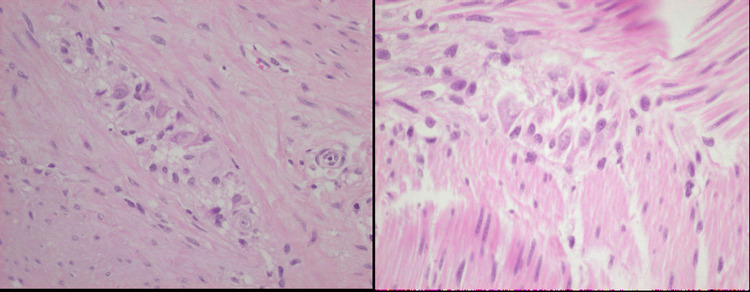
Higher magnification view showing preserved mucosal lining with small intestinal-type epithelium and a well-developed muscular layer containing nerve plexuses and ganglion cells. Areas of chronic inflammation with eosinophils and lymphoid aggregates are also evident. Hematoxylin and eosin (H&E) stain, original magnification ×40.

Microscopic analysis confirmed extramural enteric duplication in both lesions. The cyst walls were composed of mucosa, submucosa, muscularis, and serosa, extending into the mesentery. The muscularis layer exhibited a well-developed nerve plexus with ganglion cells, consistent with enteric origin. The epithelial lining varied across regions, displaying single-layered columnar epithelium, papillary morphology, and areas resembling small intestinal mucosa. In some regions, epithelial loss was noted, with replacement by chronic inflammatory granulation tissue. The cyst walls also exhibited chronic inflammatory infiltrates, including eosinophils, lymphoid aggregates, abscess foci, and foamy histiocytes, indicative of a prolonged inflammatory response (Figures 2, 3).

These findings confirm the diagnosis of extramural enteric duplication cysts with chronic inflammatory changes. The presence of well-defined intestinal wall layers and enteric neural elements supports the origin of both cysts from the gastrointestinal tract, while epithelial variability and inflammatory changes suggest a longstanding pathological process.

Although enteric duplication cysts are typically solitary, this case demonstrated two anatomically and histologically distinct lesions: a pedunculated, non-communicating cyst, and a tubular, communicating cyst associated with volvulus of the terminal ileum.

The cysts had separate mesenteric attachments, no shared lumen, and differing anatomical and structural features. These characteristics support the classification of this case as one of multiple enteric duplication cysts - a rare presentation accounting for only 1-7% of all reported cases.

## Discussion

EDCs were first described by Calder in 1733 and later by Fitz in 1884 [1]. In 1937, Ladd introduced the term “duplication of the alimentary tract”. These congenital anomalies are thought to originate during embryonic development, typically between the fourth and eighth weeks of gestation. Despite various hypotheses attempting to explain their formation, the exact etiology remains unclear. Several embryological theories have been proposed, yet none fully account for the wide range of EDC presentations, locations, and associated anomalies. One frequently suggested theory is the split notochord theory, which suggests that aberrant interactions between the notochord and endoderm lead to duplication of the gastrointestinal tract [6,7]. 

A key diagnostic feature of EDCs is their proximity to the native gastrointestinal tract, typically sharing a common epithelial lining and blood supply. These cysts may be either communicating or non-communicating with the bowel lumen. Isolated non-communicating duplication cysts can still be diagnosed histologically based on their distinct muscle wall, lining epithelium, and enteric neural components [2].

The prenatal diagnosis of enteric duplication cysts plays a crucial role in improving neonatal outcomes and optimizing perinatal management. Advances in ultrasound and fetal MRI have enhanced the early detection of these anomalies, allowing for timely intervention to prevent complications such as obstruction, volvulus, perforation, or infection. Prenatal imaging helps in differentiating EDCs from other cystic lesions, ensuring accurate diagnosis and appropriate postnatal management. 

In addition, early identification of EDCs facilitates delivery planning at specialized centers equipped with a multidisciplinary team, reducing perinatal risks and enabling prompt neonatal surgical intervention if needed. Parental counseling is also an essential aspect, providing families with information about prognosis, potential surgical needs, and postnatal follow-up. Elective surgical resection based on prenatal diagnosis often results in better outcomes compared to emergency surgeries performed for symptomatic complications [8,9]. Moreover, documenting rare prenatal cases, particularly multiple EDCs, contributes valuable insights into the embryology, clinical spectrum, and optimal management strategies for these rare anomalies.

While some EDCs remain asymptomatic, others can cause life-threatening complications, depending on their size, location, and relationship with adjacent structures. The most common complications include intestinal obstruction, perforation, hemorrhage and chronic inflammation. The presence of multiple EDCs, as seen in this case, further increases the risk of complications necessitating careful preoperative assessment and surgical planning. 

Surgical resection remains the gold standard for management, particularly in cases where obstruction, twisting, or communication with the bowel lumen is present. The choice of surgical approach depends on factors such as size, location, and presence of vascular involvement. In our case, early postnatal intervention successfully prevented potential complications such as volvulus or bowel obstruction and allowed for the preservation of normal bowel anatomy and function.

## Conclusions

This case highlights the importance of prenatal diagnosis, surgical management, and long-term follow-up in multiple enteric duplication cysts. Advances in fetal imaging have significantly improved early detection and perinatal planning, leading to better postnatal outcomes. Given their rarity and potential for serious complications, EDCs - especially multiple cysts - require multidisciplinary management, emphasizing the need for continued research to refine diagnostic criteria and surgical strategies.
